# Laparoscopic splenectomy for a wandering spleen complicating gastric varices: report of a case

**DOI:** 10.1186/s40792-014-0003-3

**Published:** 2015-01-16

**Authors:** Masanori Sato, Yuichiro Miyaki, Junpei Tochikubo, Takanobu Onoda, Norihiko Shiiya, Hidetoshi Wada

**Affiliations:** First Department of Surgery, Hamamatsu University School of Medicine, 1-20-1 Handayama Higashiku, Hamamatsu, Shizuoka 431-3192 Japan

**Keywords:** Wandering spleen, Gastric varices, Laparoscopic splenectomy, Laparoscopic splenopexy

## Abstract

Wandering spleen is a rare clinical entity, and its chronic torsion of the vascular pedicle result in splenic vein occlusion leading to gastric varices. Here, we present a case of wandering spleen complicating gastric varices in a 40-year-old female. Three-dimensional CT (3D-CT) clearly showed the disruption of the splenic vein at the origin of the vascular pedicle and collateral development of the gastric varices. The patient was electively treated with laparoscopic splenectomy. Difficulty of prediction of the splenic vein recanalization to improve the varices was the reason for the use of splenectomy versus splenopexy. The varices were successfully diminished 3 months after the surgery. After review of cases of complicating gastric varices in the literatures, splenectomy is still a secure way to treat an adult patient with wandering spleen with complicating gastric varices.

## Background

Wandering spleen is characterized by extensive mobility and displacement of the spleen from its normal position. The incidence is quite rare, with less than 0.9% in several series of splenectomies, having two peaks - in childhood and in adult females aged 20 to 40 years being of reproductive ages with suggested etiology of congenital anomalies in the development and acquired laxities of the supporting structures, respectively [1,2]. Chronic torsion of the vascular pedicle from a wandering spleen leads to splenic venous occlusion and development of collateral varicose veins, which is even rarer. Splenopexy is a favorable surgical treatment when the spleen is viable, while splenectomy is obligated to be adopted when it is infarcted [3]. Here, we present a case of wandering spleen with chronic torsion of the vascular pedicle complicating gastric varices. However, there was little guidance to determine which technique should be adopted for this specific condition. We reviewed cases of gastric varices secondary to wandering spleen in the available literature.

## Case presentation

A 40-year-old female presented to our hospital with acute onset of abdominal and back pain 2 weeks after a spontaneous abortion. She had occasionally experienced similar episodes of the abdominal symptoms, which subsided spontaneously, for 10 years after a Caesarean delivery. On further inquiry, she gave a history of exploratory laparotomy due to an idiopathic abdominal pain 20 years ago in which the ectopic spleen was identified. She denied nausea, vomiting, and tarry stools. Laboratory data were unremarkable except for a reduced platelet count of 11.1 × 10^3^/μl. Physical examination revealed a well-defined, mobile, firm, and tender 12-cm mass in her hypogastric region.

**Figure 1:**
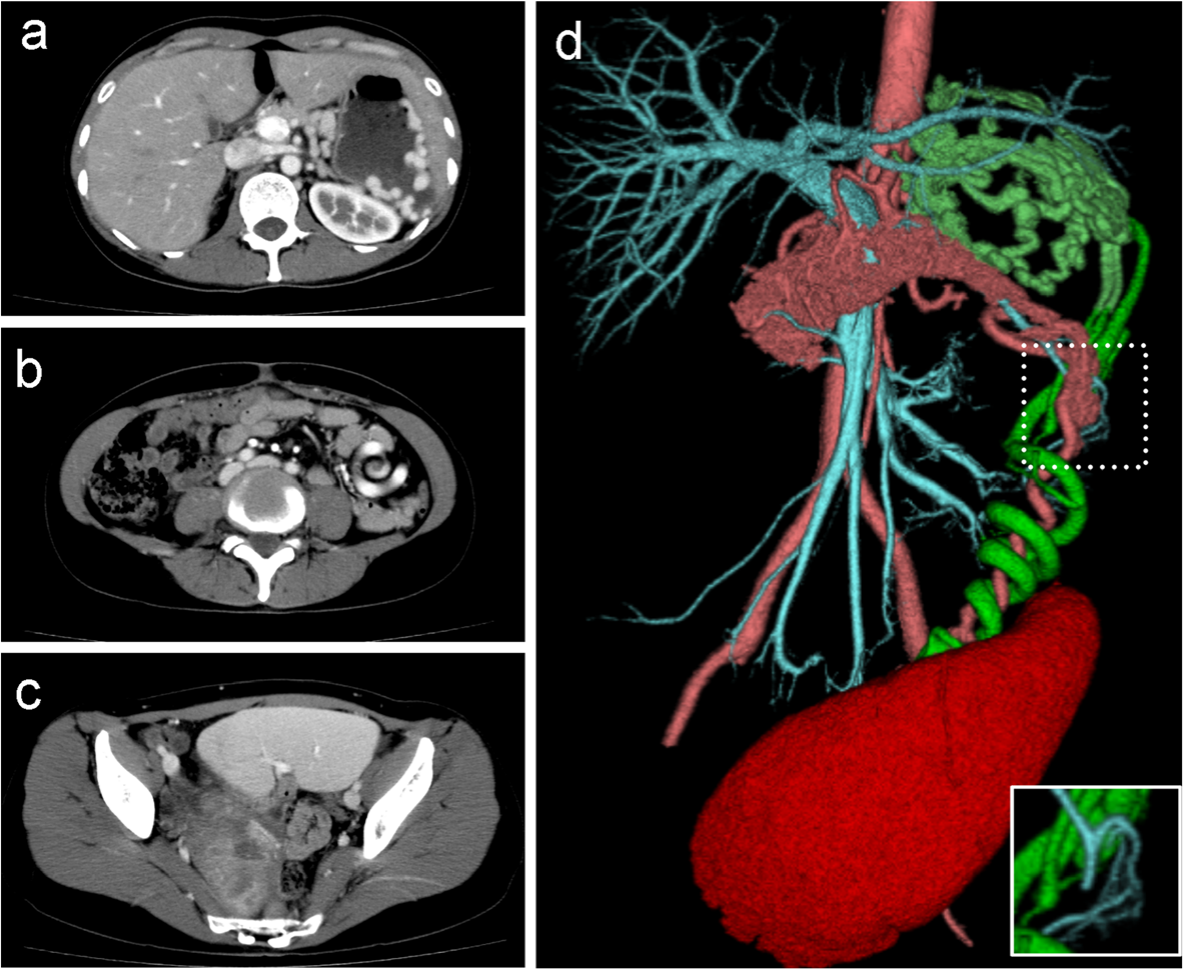
**Abdominal contrast-enhanced multidetector CT imaging. (a)** The isolated gastric varices on the gastric fornix. **(b)** The vascular pedicle showing a whirled appearance. **(c)** Displacement of the spleen in the lower abdomen with homogenous enhancement. **(d)** 3D-CT construction with vessel components. Disruption of the splenic vein at the pancreatic tail is observed in the dot square which was depicted without the arterial component in the solid square. Light blue: hepatic portal vein, splenic vein, and superior mesenteric vein. Light red: aorta, splenic artery, and small vessels in the pancreatic parenchyma. Green: collateral veins. Red: spleen.

Abdominal ultrasound examination showed a parenchymatous organ in her lower abdomen. Contrast-enhanced abdominal CT disclosed absence of the spleen in the normal position and an enlarged spleen presented in the lower abdomen. It was connected with the elongated vascular pedicle which showed a whirled appearance of splenic vessels and surrounding fat, indicating torsion of the wandering spleen (Figure 1a-c). Homogenous enhancement of the spleen represented absence of splenic infarction. Engorged collateral veins from the pedicle continued into the gastric fornix to form isolated gastric varices, suggesting her chronic history. A construction of the 3D-CT revealed that the splenic vein within the splenic arterial helix was disrupted at the pancreatic tail (Figure 1d). Upper gastrointestinal endoscopy revealed isolated gastric varices without any red signs.

**Figure 2:**
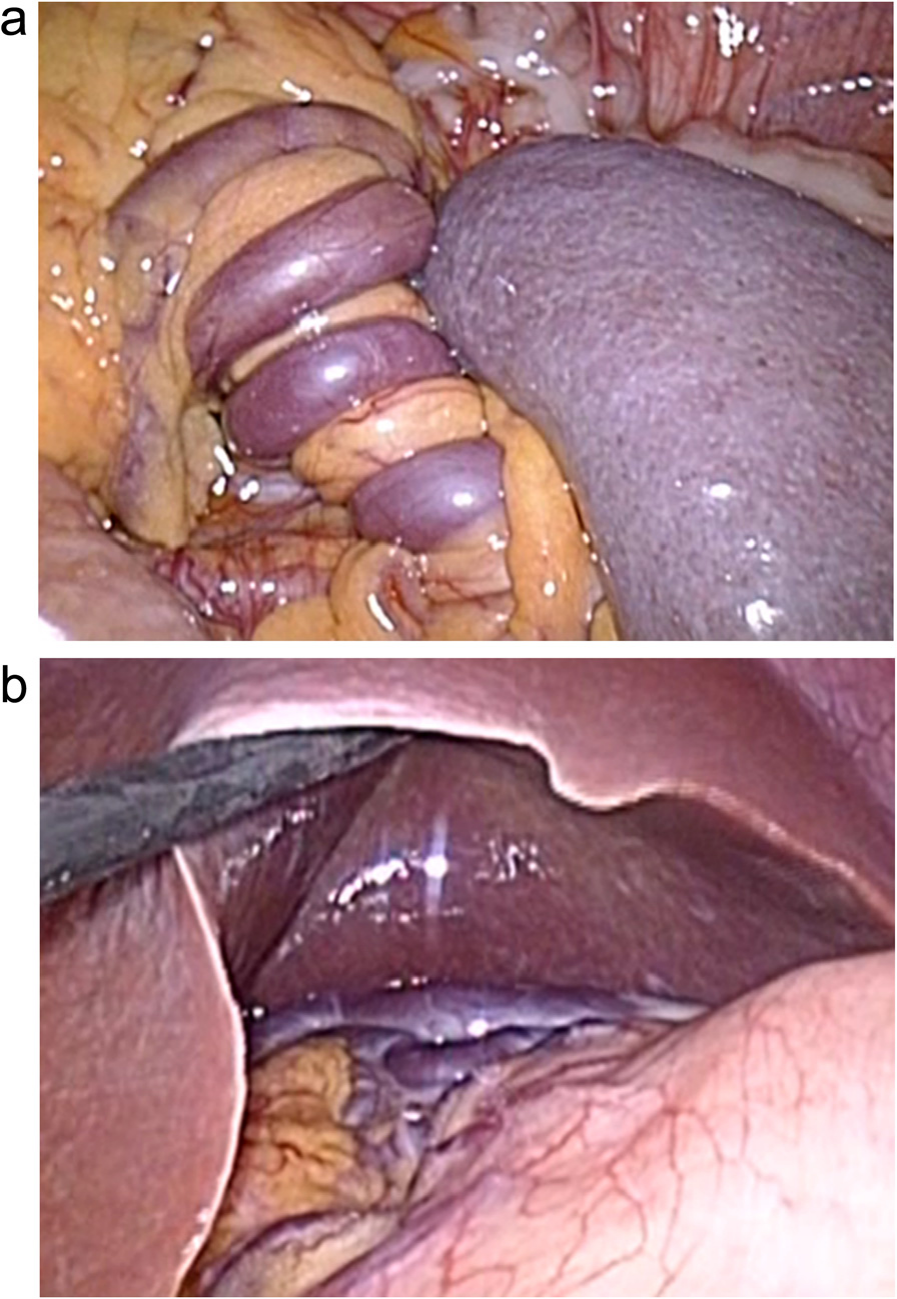
**Laparoscopic view of the vascular pedicle and gastric varices. (a)** The elongated vascular pedicle was twisted left 1,440° wrapping with the helically engorged collateral veins beside the wandering spleen. **(b)** The engorged isolated gastric varices on the gastric fornix.

Elective laparoscopic surgery was done with placement of three trocars at the sub-umbilicus, suprapubic, and the middle of the upper abdomen 2 weeks after the consultation. Intraoperatively, the enlarged spleen was found in the lower abdomen, and there was no anchorage to the retroperitoneum and left phrenicus. The gastrosplenic ligament and splenic hilus were fused and elongated to suspend the spleen and turned 1,440° (Figure 2a). Several engorged veins on the pedicle continued into the gastric fornix and appeared vulnerable to rupture with surgical manipulation (Figure 2b). Splenectomy was performed by transection of the pedicle with a surgical staple. We selected splenectomy instead of splenopexy because of the difficulty of intraoperative prediction for the splenic vein recanalization and the improvement of the varices after detorsion. The histopathologic examination showed no infarction with the parenchyma or any thrombosis and physical obstruction in the splenic vein. The patient received pneumococcal vaccines 3 weeks after surgery. Three months later, the woman was doing well and the gastric varices were diminished on CT examination.

## Discussion

Wandering spleen is a rare clinical entity, and its complication with gastric varices is even rarer [1,4]. The gastric varices most likely arose from splenic vein occlusion secondary to chronic torsion of the vascular pedicle, leading to retrograde congestion of the short gastric and left gastroepiploic veins. As the patient was not suffering from hematemesis or melena in our case, presence of the gastric varices was detected by the CT image. The contrast-enhanced CT was quite useful, not only for a definitive diagnosis and assessment of splenic viability but also accompanying collateral venous expansion. Especially by means of 3D-CT image, the disruption of the splenic vein at the origin of the vascular pedicle could be recognized.

The definitive treatment for wandering spleen is surgery, since non-operative treatment is associated with a complication rate as high as 65% [5]. Splenectomy has long been the conventional treatment for wandering spleen. Currently, splenopexy becomes a favorable alternative especially in pediatric patients, without involving splenic infarction or venous thrombosis, to avoid the risk of overwhelming post-splenectomy sepsis [3]. In cases complicated by gastric varices, meanwhile, elimination of the gastric varices is another purpose of the treatment in addition to symptom relief and relapse prevention. Regression of the varicose vein would be expected once the splenic vein was recanalized [6], but it is unpredictable pre- or intraoperatively. A recent multicenter study reported a complication of splenic ischemia after splenopexy with a mesh in 60% of cases [2]. It was speculated that the elongated vascular pedicle might kink after splenopexy to impair the blood flow.

There were few reports discussed about appropriate surgical management with chronic torsion of the vascular pedicle complicating gastric varices. We reviewed cases of gastric varices secondary to wandering spleen including our case in the literatures (Table 1). Of 14 cases in total, 10 patients (71%) were adult, being consistent with prolonged suffering from chronic torsion of the vascular pedicle. Gastrointestinal bleeding presenting hematemesis and/or melena were presented in nine patients. The region of the varices was not extended to the esophagus but confined to the stomach in all cases. No cases were complicated with total splenic ischemia. Thrombosis in the splenic vein was reported intraoperatively or histologically in three cases. Splenectomy was selected in the 13 cases (93%), and the outcomes were satisfactory as the varicose veins were not detected with endoscopy or CT in all case follow-ups. Splenectomy was selected for two young patients complicating with not only gastric but also portal and mesenteric varices [6]. However, only a single case reported by Wani S et al. underwent detorsion and splenopexy in which elimination of the gastric varices was confirmed with the follow-up endoscopy [4]. Thus, splenectomy was employed in almost all cases. Therefore, it remains unclear whether the collateral varicose veins will be reduced or not by splenopexy.

**Table 1:** **Literature review of case reports on wandering spleens complicating gastric varices**

**Author**	**Year**	**Age/sex**	**GI bleeding**	**Ischemia**	**Thrombosis**	**Operation**	**Postoperative varicose veins**
Smulewicz JJ [7]	1975	35/F	−	−	+	Open splenectomy	NA
Inukai H [8]	1977	23/M	+	−	−	Open splenectomy	NA
Daneshgar S [9]	1980	14/F	+	NA	−	Open splenectomy	Not detected
Sorgen RA [10]	1980	14/F	+	−	−	Open splenectomy	NA
Angeras U [11]	1984	41/F	+	−	−	Open splenectomy	Not detected
Sugishita T [12]	1987	22/F	−	−	−	Open splenectomy	Not detected
Habib E [13]	2001	23/F	+	Partial	+	Open splenectomy	NA
Gilman RS [14]	2003	25/F	+	−	−	Open splenectomy	Not detected
Tan HH [15]	2007	28/F	−	Partial	−	Open splenectomy	NA
Singla V [16]	2008	45/F	+	−	+	Open splenectomy	Not detected
Wani S [4]	2008	25/F	+	−	−	Open splenopexy	Not detected
Irak K [17]	2011	55/F	+	−	−	Open splenectomy	Not detected
Zarroug AE [6]	2013	16/F	−	−	−	Laparoscopic splenectomy	Not detected
Zarroug AE [6]	2013	12/F	−	−	−	Open splenectomy	Not detected
Sato M	2014	40/F	−	−	−	Laparoscopic splenectomy	Not detected

NA, not available; GI bleeding, gastrointestinal bleeding.

## Conclusions

Splenopexy would be an appropriate surgical option when recanalization of the splenic vein was certainly established. However, splenectomy is still a secure way to cure the gastric varices secondary to wandering spleen.

## Consent

Written informed consent was obtained from the patient for publication of this case report and any accompanying images. A copy of the written consent is available for review by the Editor-in-Chief of this journal.
